# Soluble Graphene Nanosheets for the Sunlight-Induced Photodegradation of the Mixture of Dyes and its Environmental Assessment

**DOI:** 10.1038/s41598-019-38717-1

**Published:** 2019-02-21

**Authors:** Gunture, Anupriya Singh, Anshu Bhati, Prateek Khare, Kumud Malika Tripathi, Sumit Kumar Sonkar

**Affiliations:** 1 Department of Chemistry, Malaviya National Institute of Technology, Jaipur, Jaipur, 302017 India; 2 Department of Bio-nanotechnology, Gachon University, Gyeonggi-do, South Korea

## Abstract

Currently, the air and water pollutions are presenting the most serious global concerns. Despite the well known tremendous efforts, it could be a promising sustainability if the black carbon (BC) soot can be utilized for the practical and sustainable applications. For this, the almost complete aqueous phase photodegradation of the three well-known organic pollutant dyes as crystal violet (CV); rhodamine B (RhB); methylene blue (MB) and their mixture (CV + RhB + MB), by using water-soluble graphene nanosheets (wsGNS) isolated from the BC soot under the influence of natural sunlight is described. The plausible mechanism behind the photocatalytic degradation of dyes and their mixture has been critically analyzed via the trapping of active species and structural analysis of photodegraded products. The impact of diverse interfering ions like Ca^2+^, Fe^3+^, SO_4_^2−^, HPO_4_^2−^, NO_3_^−^, and Cl^−^ on the photodegradation efficiency of wsGNS was also investigated. Importantly, the environmental assessment of the whole process has been evaluated towards the growth of wheat plants using the treated wastewater. The initial studies for the fifteen days confirmed that growth of wheat plants was almost the same in the photodegraded wastewater as being noticed in the control sample, while in case of dyes contaminated water it showed the retarded growth. Using the natural sunlight, the overall sustainability of the presented work holds the potential for the utilization of pollutant soot in real-practical applications related to the wastewater remediation and further the practical uses of treated water.

## Introduction

The ever-growing desire to improve the quality of human lifestyle significantly promoted the rapid industrialization and urbanization^1–3^. Primarily, associated with the accelerating advancement of the automotive industrialization, which can directly link to the release of dirty-dangerous pollutant black soot as black carbon (BC)^4–7^ particulate matter in the environment. BC is closely related with global warming and at present is continuously deteriorating the environmental and human health^7,8^. Along with air pollutions, industrialization have also brought the another important concern related to the water pollutions^9^. So, the most demanding aggravated concerns of the present world is to significantly reuse the waste products (it could also be the dangerous-dirty-BC)^10^ and the treatment of wastewater^11,12^. From the standpoint of the overall environmental health, the discharge of the BC^7^ in air and the effluents of industrial wastewater^13–15^ (containing hazardous, carcinogenic and non-biodegradable organic dyes) in the water-bodies are unceasingly deteriorating the ecological balance^16,17^, and causes many serious diseases^18–20^. At present, few groups have explored the recent-promising approaches related to the adaptation of pollutant soot as freely available carbon precursor for the synthesis/isolation of the value-added nano-carbons^21–29^. Such as carbon dots (CD)^23^, graphene nanosheets (GNS)^24,25,27^, single-walled carbon nanotubes (SWCNT)^26^, carbon nanoparticles^22^ used for the diverse applications^22,23,27^ including the photodegradation of the pollutant dyes^24,25^. In the same context, the visible-light photocatalysis using the nano-carbons, metal-based carbon nano-materials^30–34^ and its composites^31,35–43^ has attracted the widespread attention, because of its interesting applicative prospects in the field of the water remediation^40,41,44^. Sunlight-induced dye degradation exhibits high efficiency along with the ability to use the most renewable and sustainable source of energy as sunlight^45^, hence can offers a feasible approach to overcome the degree of water pollutions.

Presently, the graphene^46,47^ and graphene-based nano-structures^37,42^ have drawn more and more attention due to their many advantageous features like high optical absorption, fast charge carrier mobility, high conductivity, non-toxicity, corrosion resistance, the unique surface properties and environmental acceptability^48^. Although few milestones have already been documented for enhancing photocatalytic efficiency of the metal-based^49–51^ or metal-graphene based nanostructures for the degradation of organic pollutant^52–55^, but their fabrications methods inevitably include the tedious, and complex process^44,56–58^. Along with this to tune the band-gap of graphene based nanostructures, additional strategies such as metal/heteroatom doping, composite fabrication and surface functionalization were further required^31,40,52,59^. additionally, the above mentioned reports were mostly deal with the photodegradation of the single component of organic dyes^32,41,44,52,60–63^. As per the general consideration, the effluents of industrial wastewater are being composed of the complex system, containing the combination of dyes. But only a little attention has been paid to remediate wastewater containing mixture of dyes^64–69^. For this, the exploration of a facile, cost-effective and sustainable approach for the synthesis of graphene-based nanostructures having the desirable light response is crucial for the application in photocatalysis. As they require a superior charge separation efficiency and a broad photoresponsive range^70^. Under the presence of sunlight, the same can be provided by the water-soluble graphene nanosheets (wsGNS), isolated from the BC possessing the advantageous efficiency to work as a photocatalytic material^24,25^. Moreover, based on the few reports posing the controversies regarding the observance of acute toxicity due to generation of toxic byproducts even after complete degradation of dyes^71–74^. The present time demands a strong requirement of environmental assessment of the whole process to rule out the possible risk and maintain the ecological balance, and being smoothly used for the real life applications.

The present finding describes a simple and feasible approach related to the utilization of the pollutant soot as a low-cost, easily available precursor for the isolation of wsGNS. wsGNS was further utilized as photocatalyst for the complete photodegradation of three individual dyes like crystal violet (CV), rhodamine B (RhB) and methylene blue (MB) and their mixture (CV + RhB + MB) under the natural sunlight irradiation. The potential of the wsGNS were further investigated under the presence of common interfering ions/substances^66^. In continuation of earlier studies^24,25^, the work described here is based on the simple idea, related to the photodegradation of the pollutant material (degradation of mixture of dyes) from the pollutant material (BC). Importantly, in addition to the aqueous phase photocatalysis only, the treated wastewater is further being utilized for the environmental risk assessment. In this context, growths of wheat (*Triticum aestivum*) plants were assessed with the dye-polluted water before and after photocatalytic degradation, including the control to evaluate the environmental applicability of the treated wastewater. Initial results are in favor that treated wastewater could be used for growing the plants that can maintain the ecological balance of the required water.

## Results and Discussion

**Figure 1:**
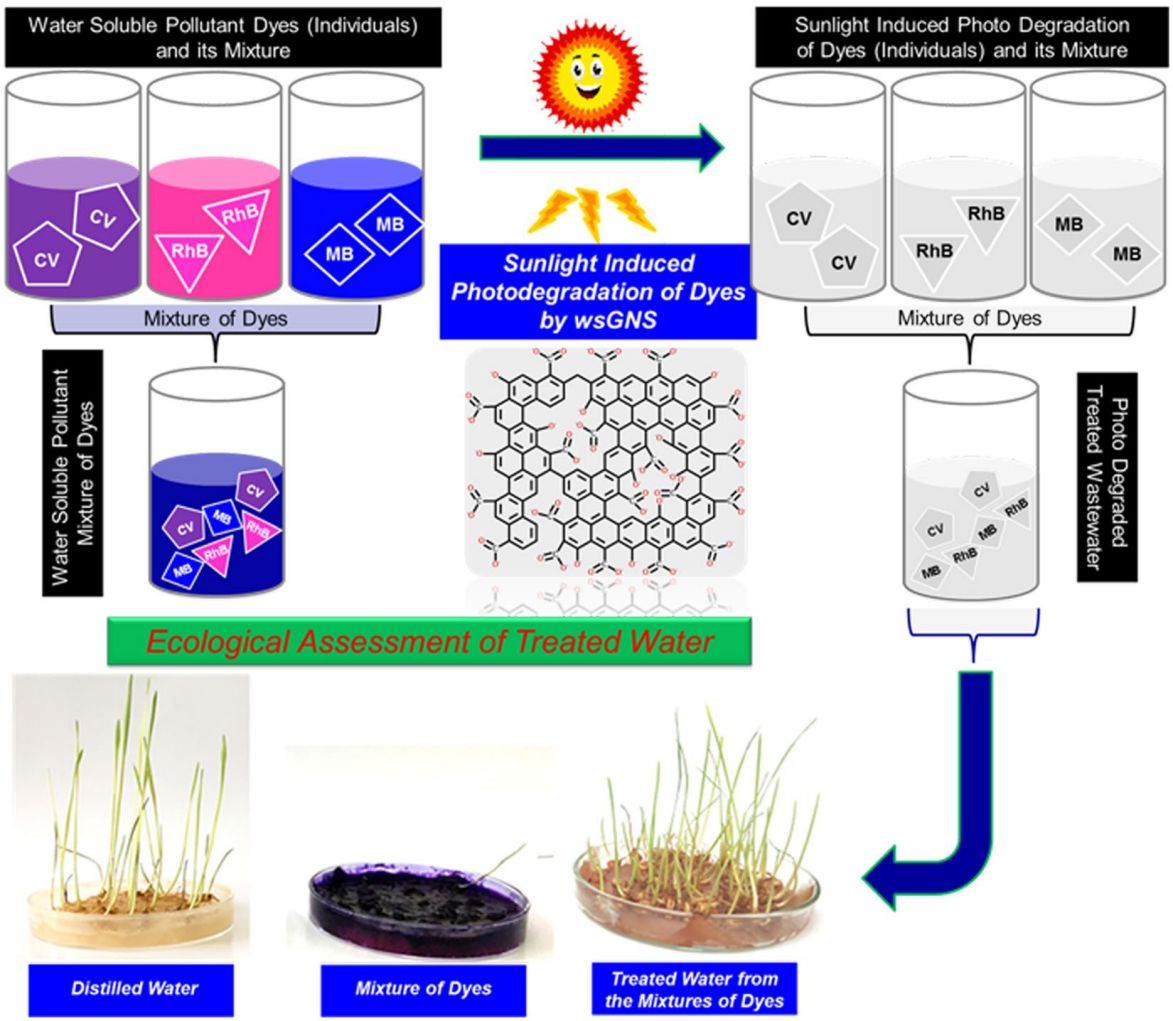
Schematic representation illustrating the application of the wsGNS for the photodegradation of the three different dyes and their mixture under the presence of sunlight. Further, the treated wastewater was being employed for growing the wheat plants.

At present, most of the photocatalytic materials are showing their selectivity towards the photodegradation of the specific dye only. Therefore, for the practical applicative prospects just target the single pollutant dye has not been a sufficient feasible approach. For the same, the photocatalytic performance of the pollutant soot isolated wsGNS^24,25^ was extended concerning the photocatalytic degradation of the three different individual model dyes as CV, RhB, MB and their mixture (CV + RhB + MB) under the influence of the natural sunlight. A simpler schematic methodology described in Fig. 1, illustrates the significant usage of BC derived wsGNS as a photocatalytic material under the influence of natural sunlight for the photodegradation of three different dyes as CV, RhB, MB and their mixture (CV + RhB + MB). As well, supports the sustainability of the overall process, concerning the reuse of the treated wastewater for growing the wheat plants.

### Microscopic and Spectroscopic characterization

**Figure 2:**
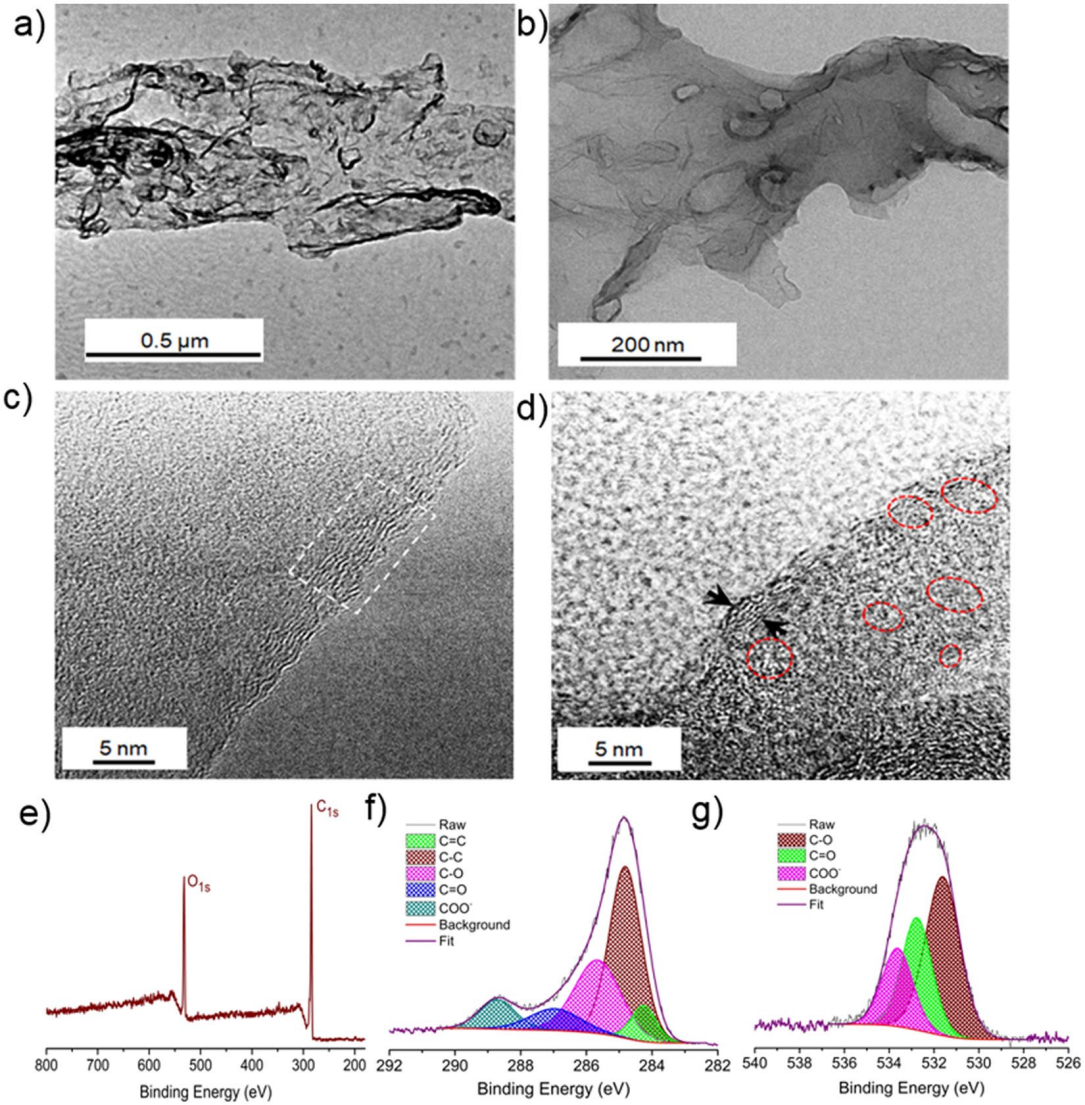
**(a**,**b)** Low-resolution TEM images of wsGNS; **(c**,**d)** HRTEM image of wsGNS show existence of few-layered graphene sheets with surface defects. **(e)** A full survey scan XPS analysis of wsGNS along with its corresponding short scan; **(f)** C1s; and **(g)** O1s.

Transmission electron microscopy (TEM) was used to analyse the morphology of wsGNS (Fig. 2(a)), which displays the randomly oriented layers of wsGNS with wrinkled and crumpled surface morphology. High magnification TEM image (Fig. 2(b)) shows the existence of mutil-layered wsGNS. The morphological insights of the wsGNS is shown in Fig. 2(c) confirm the lattice fringes, existence of multi-layers graphene (white box of Fig. 2(c)). Figure 2(d) shows the presence of differentially oriented graphitic patches (marked by red circles and black arrows shows the multilayers), which are being generated via random breaking of sp^2^ hybridization of the carbon atoms by the vigorous oxidation. The interplanar spacing of ~0.34 nm as shown in Fig. 2(d) could be assigned to the (002) plane of the few-layer wsGNS^27^.

X-ray photoelectron spectroscopy (XPS) measurements were used to describe the surface elemental composition and the nature of surface functional groups on the wsGNS^24^. XPS spectrum (Fig. 2(e)) of wsGNS shows the two characteristic prominent peaks at ~284.4 eV and ~532.1 eV for the C_1s_ and O_1s_ respectively. The high resolution C_1s_ XPS spectrum (Fig. 2(f)) was deconvoluted into five states of C_1s_ as C=C, C-C, C-O, C=O, and COO^−^ corresponding to the binding energy of 284.2, 284.8, 285.6, 286.7 and 288.8 eV respectively. Figure 2(g), shows the high resolution O_1s_ XPS spectrum, which exhibits the presence of three peaks at 531.3, 532.5 and 533.7 eV corresponding to C-O, C=O, and COO^−^ respectively. Higher in the oxygen content confirms the incorporation of high-degree of the negative organic functional moieties as hydroxyl and carboxyl groups over the surface of wsGNS and termed as the surface defects. The high-density surface-defects have the strong ability to facilitate the photocatalytic efficiencies of the wsGNS^27^, for the photodegradation of the dyes and their mixture in the influence of sunlight. The confirmation of the graphene sheets and structural information over disorder and functionalization were further achieved by Raman spectroscopy^25,75^. The Raman spectrum (Fig. S1(a)) of wsGNS exhibits characteristic broad disorder induced D band at ~1345 cm^−1^, graphitic G band at ~1614 cm^−1^ and a overtone 2D band at ~2703 cm^−1^. The D band arises due to the breathing modes of sp^3^ atoms in rings, while G band attributed to the bond stretching of sp^2^ atoms in both rings and chains. The 2D band is referred as a second order D band arises from a two-phonon lattice vibrational process, and is the second most prominent band (after G band) for graphitic structures. The high I_D_/I_G_ ratio (~1.48) suggesting the higher density of defects in the form of oxygen containing functional groups. High-density surface functionalization has been validated by the FT-IR spectrum of wsGNS^25^ (Fig. S1(b)) which display broad absorption band at ~3432 cm^−1^ related to O-H stretching vibrations signifying presence of hydroxyl groups, sharp absorption peak due to presence of carbonyl functional groups appears at ~1714 cm^−1^, and peak at ~1624 cm^−1^ corresponds to C=C stretching. Another peak at 1233 cm^−1^ relates to C-O stretching vibrations. The X-ray diffraction (XRD) pattern of wsGNS (Fig. S1(c)) exhibits three prominent diffraction peaks, a sharp and intense peak at ~24.84°, a broad peak at ~42.35° and a sharp and weak peak at ~72.77° corresponds to the reflections from the (002), (100) and (110) graphitic planes.

### Photocatalytic dye degradation under sunlight irradiation

**Figure 3:**
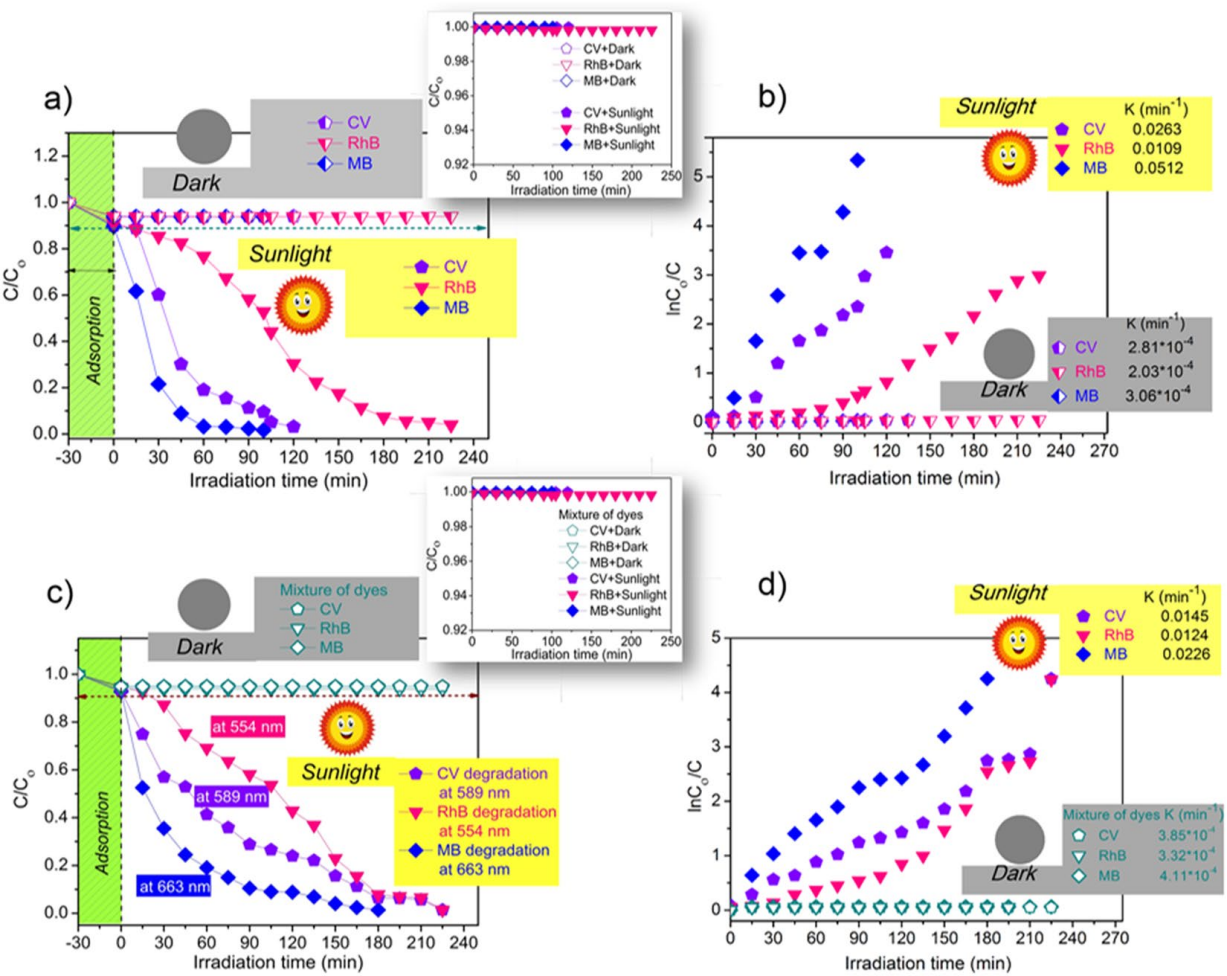
(**a**) Extent of plot of (C/C_ο_) for the individual dyes (CV, RhB and MB) with wsGNS in dark and sunlight (with inset showing photocatalytic degradation of the respective dyes in the absence of wsGNS); with their **(b)** respective plot of ln(C_ο_/C); **(c)** Extent of plot of (C/C_ο_) (with inset showing photocatalytic degradation of the mixture of dyes in the absence of wsGNS) of a mixture of dyes with wsGNS in dark and sunlight; with their **(d)** respective plot of ln(C_ο_/C).

The photocatalytic degradation efficiency of wsGNS is displayed in Fig. 3 for the freshly prepared three different dyes and their mixture (all the individual dyes are having the concentration of 20 ppm and for the mixture it contains the 20 ppm of the each dye)^64,65,67,68^. An adsorption-desorption balance was attained between the wsGNS and dyes (CV, RhB, and MB) and their mixture (CV + RhB + MB) for the initial 30 min via stirring. In the dark (as a control experiment) at the same experimental conditions, prior to the process of the photocatalytic degradation. It was observed that ~11% CV, ~10% RhB, ~11% MB and ~12% of their mixture (CV + RbB + MB) were adsorbed on the wsGNS in 30 minutes. The photocatalytic degradation of dyes was accessed by monitoring the relative change in concentration with time, concerning the rate of decolorization. The change in intensity of characteristic peaks using UV-Vis absorption spectroscopy were recorded for CV, RhB, and MB at 589 nm, 554 nm, and 663 nm respectively. Concerning the interference of absorbance with photodegradation the control set of experiments (marked as Dark (Fig. 3(a,c)) were being conducted in absence of sunlight with wsGNS. No significant changes in the concentration of dyes and their mixtures were obtained, after attaining the adsorption-desorption equilibrium. As well, another control test for the photocatalytic degradation of the respective dyes and their mixture in the absence of wsGNS could almost be overlooked (as shown in the inset of the Fig. 3(a,c), which confirms the high photostability of dyes under the presence of sunlight. From the photodegradation results, as expected, the different rate constant for the photodegradation of different dyes could be attributed to their difference in the chemical structures. In the presence of wsGNS, MB showed the fastest photodegradation as within 100 min, ~99% of MB degraded. CV showed moderate degradation and degraded ~99% within the 120 minutes, while the RhB takes a bit longer time ~225 minutes (might be because of the complex organic framework in comparison with CV and MB) for ~99% of its photodegradation as shown in Fig. 3(a). In the same panel, Fig. 3(b) shows the apparent rate constant related to the photodegradation of MB, CV and RhB as 0.0512 min^−1^, 0.0263 min^−1^ and 0.0109 min^−1^ respectively. Concerning the photocatalytic degradation of the mixture of dyes (CV + RhB + MB), the absorption spectrum shows the appearance of three different peaks (589 nm, 554 nm, and 663 nm) related to respective dyes as CV, RhB, and MB. The clarity in differing the three peaks is very much advantageous for the present study. The photodegradation efficiency (Fig. 3(c)) of respective dyes from the mixture (CV + RhB + MB) was being analyzed based on their respective absorbance value (λ_max_ values of 589 nm, 554 nm, and 663 nm). Similar to the individual dyes, the mixture of dyes displays the progressive decrease in its concentration by wsGNS under the presence of sunlight (Fig. 3(c)). The degradation rate of dyes in mixture were found to be 180 min for the MB, and for the case of CV, and RhB it was ~225 min. The rate constants for CV, RhB, and MB were observed as 0.0145 min^−1^, 0.0124 min^−1^ and 0.0226 min^−1^ respectively (Fig. 3(d)) in mixture based on their photodegradation efficiency observed at their respective λ_max_ values. The decrease in rate constant in the mixture of dyes, compared to the individual dyes can be because of the competitive occupancy of optically active centers in-between the photodegradation process over the surface of the wsGNS. The detailed UV-Vis absorption study as shown in Fig. S2(a–e) (supporting information (SI)) shows the relative change in concentration of respective dye and their mixture (decrease in the color intensity) with time under the presence of sunlight. Fig. S2(d) shows the absorption spectra of the mixture of dyes (CV + RhB + MB) easily differentiated based on three separate peaks in the mixture because of absorbance associated with CV, RhB, and MB.

The sunlight responsive properties of wsGNS were supported by the UV-Vis diffuse reflectance spectroscopy (DRS). The near edge absorption of wsGNS appears at ~260 nm as shown in Fig. S3(a) which attributes to the band gap of wsGNS. The Tauc plot^76–81^ ((αhυ)^2^ vs hυ) (Fig. S3(b)) have been displayed the band gap of wsGNS, which was found to be ~3.43 eV before the photodegradation of the mixture of dyes while after the photodegradation there was a bit decrease in the band gap of wsGNS ~3.16 eV. This might be because of the adsorption of some fragmented dye molecules over the surface of wsGNS.

### Trap Study

**Figure 4:**
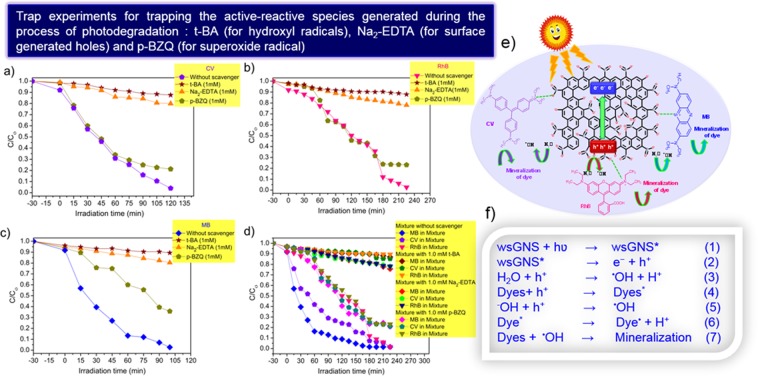
Effect of scavengers t-BA, Na_2_-EDTA, and p-BZQ for hydroxyl, holes, and superoxide radicals respectively on the degradation performance of **(a)** CV, **(b)** RhB, **(c)** MB and **(d)** on the mixture of dyes. **(e)** Schematic illustration showing degradation of different dyes using wsGNS under sunlight irradiation; **(f)** with their respective suggested pathways.

To explore the active moieties involved in photocatalytic degradation of dyes^34,82–87^ under direct sunlight irradiation radical scavengers were introduced to trap specific reactive species. A simpler trap experiment has been performed based on the scavenging properties of disodium ethylene diaminetetraacetate (Na_2_-EDTA) for trapping of the surface generated holes (h^+^), tert-butyl alcohol (t-BA) for trapping the hydroxyl radicals, and the para-benzoquinone (p-BZQ) for the trapping of superoxide (O_2_^−.^) radicals. In all the cases concentrations of scavengers were fixed at 1 mM, and their effects were observed for the change in concentration of dyes (in terms of C/C_o_) as displayed in Fig. 4(a–c) for CV, RhB, and MB respectively. As perceived in Fig. 4(a–c) the photodegradation process in comparison to control, where no scavengers were used considerably inhibited in the presence of t-BA and Na_2_-EDTA implying that hydroxyl radicals and h^+^ were significantly participating in the process of the photodegradation. The degradation efficiencies of dyes, on the addition of t-BA decrease from 99% to 13%, 99% to 12% and 99% to 11% for CV, RhB, and MB respectively. Similarly, the addition of Na_2_-EDTA reduces the degradation efficiency from 99% to 21%, 99% to 20% and 99% to 21% for CV, RhB, and MB respectively. Contrary to additions of t-BA and Na_2_-EDTA, a very slight decrease in the photodegradation efficiency of dyes were observed with the addition of p-BZQ, supporting that O_2_^−.^ are not actively participating in photodegradation process. Almost similar trends were observed for the mixture (CV + RhB + MB) of dyes (Fig. 4(d)).

Based on the results from the trapping experiments, a schematic of the possible mechanism for the photocatalytic degradation of the dyes and their mixture by wsGNS under the direct sunlight irradiation is proposed in Fig. 4(e). wsGNS get photosensitized during the irradiation of sunlight, and the electron-hole pairs are generated (eqs 1 and 2 from Fig. 4f). The trapped photoexcited electrons were lead to higher charge transfer and electron-hole pair separation efficiency, while the photo-induced holes accumulated on the surface adsorbed water molecule would initiate the generation of hydroxyl radical which might directly react with dyes (eqs 3 and 4 from Fig. 4f). Hydroxyl radical, as the predominant species attacked on dye molecules and contribute towards the effective photodegradation of respective dyes and its mixture (eqs 5–7 from Fig. 4f).

### NMR investigation of degraded products of CV, RhB and MB and their mixture (CV + RhB + MB)

**Figure 5:**
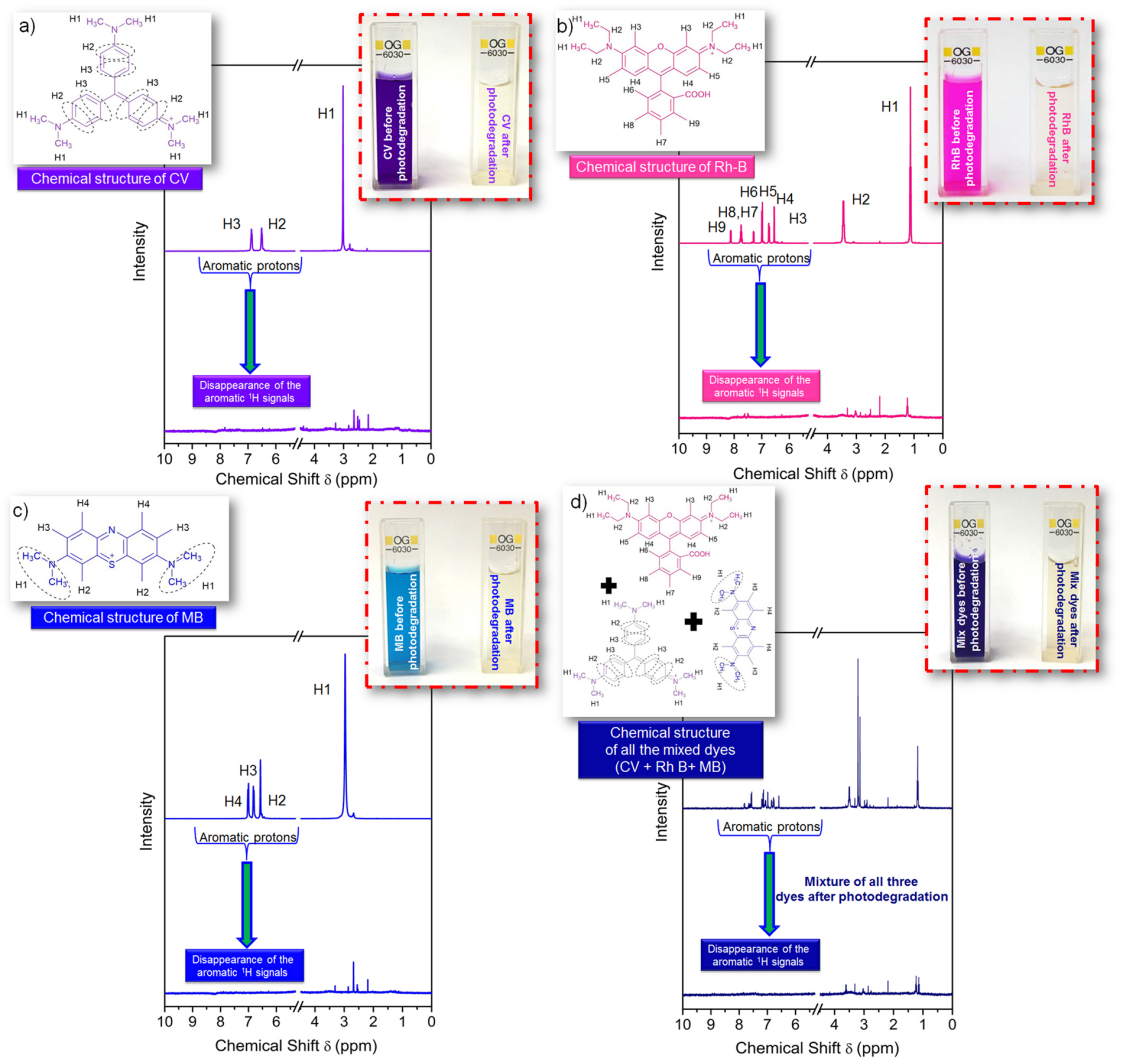
The chemical structure of **(a)** CV; **(b)** RhB; **(c)** MB; and **(d)** Mixture of dyes; including with their respective ^1^H NMR spectra, before and after their photodegradation by wsGNS. Inset of all figure **(a**–**d)**, shows the digital images of respective dye **(a**–**c)** and their mixture **(d)** before and after the photodegradation.

During the process of photodegradation the formation of smaller fragments of respective dyes were been analyzed by a straightforward comparative ^1^H-NMR analysis for the control sample (individual dye and their mixture) *versus* the photodegraded products of respective dyes and their mixture (CV + RhB + MB). Figure 5(a–d) shows the comparative aqueous phase ^1^H NMR analysis of the control dyes and their mixture (CV + RhB + MB) with their respective photodegraded products. The ^1^H NMR spectra of CV, RhB, and MB (Fig. 5(a–c)) were taken before and after the photodegradation of dyes (samples collected from the supernatant from the pool of dye-wsGNS system were being dried and dissolved in D_2_O for the ^1^H NMR analysis). After the time interval of the four hours of sunlight irradiations; the samples were collected for all CV, RhB, MB and their mixture (CV + RhB + MB), for NMR analysis^24,25^. Figure 5(a), shows the proton peaks associated with the aliphatic and aromatic region of the CV. ^1^H NMR (400 MH_Z_, D_2_O): δ (ppm) 3.03 (s, 18 H), 6.50 (d, J = 7.6 H_Z_, 6 H), 6.87 (d, J = 7.6 H_Z_, 6 H). Over a comparative analysis after photodegradation experiment for four hours of sunlight irradiation on the pool of CV-wsGNS mixture, it shows the breakage of the complete aromatic framework of the used dye (disappearance of the signals associated with the aromatic protons). As well, the ^1^H NMR spectrum of the photodegraded CV showed the appearance of new smaller aliphatic peaks at δ 2.15–3.28 ppm (m, fragmented hydrocarbons), which can be directly related to the disintegration/mineralization of the original aromatic organic framework of CV molecules into the smaller aliphatic fragments. Similarly, for the other two dyes (RhB and MB), the same had been observed. The proton signals from the RhB (Fig. 5(b)) were divided into the two regions; ^1^H NMR (400 MH_Z,_ D_2_O): δ (ppm) 1.12 (t, J = 7.2, 12 H), 3.39–3.50 (m, 8 H), 6.55 (d, J = 2 H_Z,_ 2 H), 6.74 (dd, J = 2 H_Z_, J = 9.6 H_Z_, 2 H), 6.69 (d, J = 9.6 H_Z_, 2 H), 7.29 (d, J = 7.2 H_Z_, 2 H), 7.71–7.79 (m, H,H), 8.12 (d, J = 8, 1 H). After the photodegradation, signature proton signals from RhB does not appear as before degradation, as well as almost the disappearance of the intense aromatic signals and the emergence of the few aliphatic protons δ 1.23–3.31 ppm (m, fragmented hydrocarbons) is in support of the disintegration of the complex organic framework of the RhB molecules into smaller aliphatic components. Likewise, in the case of MB, before (Fig. 5(c)), ^1^H NMR (400 MH_Z_, D_2_O): δ (ppm) 2.97 (s, 12 H), 6.57 (s, 2 H), 6.82 (d, J = 8.8 H, 2 H), 7.01(d, J = 8.8 H_Z_, 2 H), and after the photodegradation, showed its dissociation into the smaller hydrocarbons (δ 2.19 ppm to 3.31 ppm) in the aliphatic region and the aromatic signals were disappeared entirely. Similar to the individual dyes, the photodegradation of the mixture of dyes (CV + RhB + MB) has also been analyzed by the NMR. A similar result was observed for the mixture of dyes in (Fig. 5(d)) which shows a comparative NMR spectrum of the mixture of dyes; before and after the photodegradation experiment. The proton signals associated with the mixture is of now becomes a complex system of the organic molecule, so the individual assigning of the proton signals is being excluded here. But after the photodegradation (Fig. 5(d)) a complete change in the proton signals; before and after the photodegradation is being observed. After the photodegradation, the disappearance of aromatic protons has strongly advocated the disintegrations of the complex organic frameworks of the mixture of dyes. The NMR analysis shows the strong influence of wsGNS under the presence of sunlight for the photodegradation applications of the pollutant dyes and their mixture (CV + RhB + MB).

### Regeneration recycling study

Addition to the photodegradation efficiency, the recycling ability of a photocatalyst is an important parameter to define the sustainability of the new proposed process. The reusability performance of the wsGNS employed up to the four cycles under sunlight irradiation towards a mixed dye solution as shown in Fig. S4. After the four cycles, there is the only loss of ~22% in the degradation efficiency. The decrease in efficiency could be attributed to loss of material during recycling.

### Effects of interfering substances

**Figure 6:**
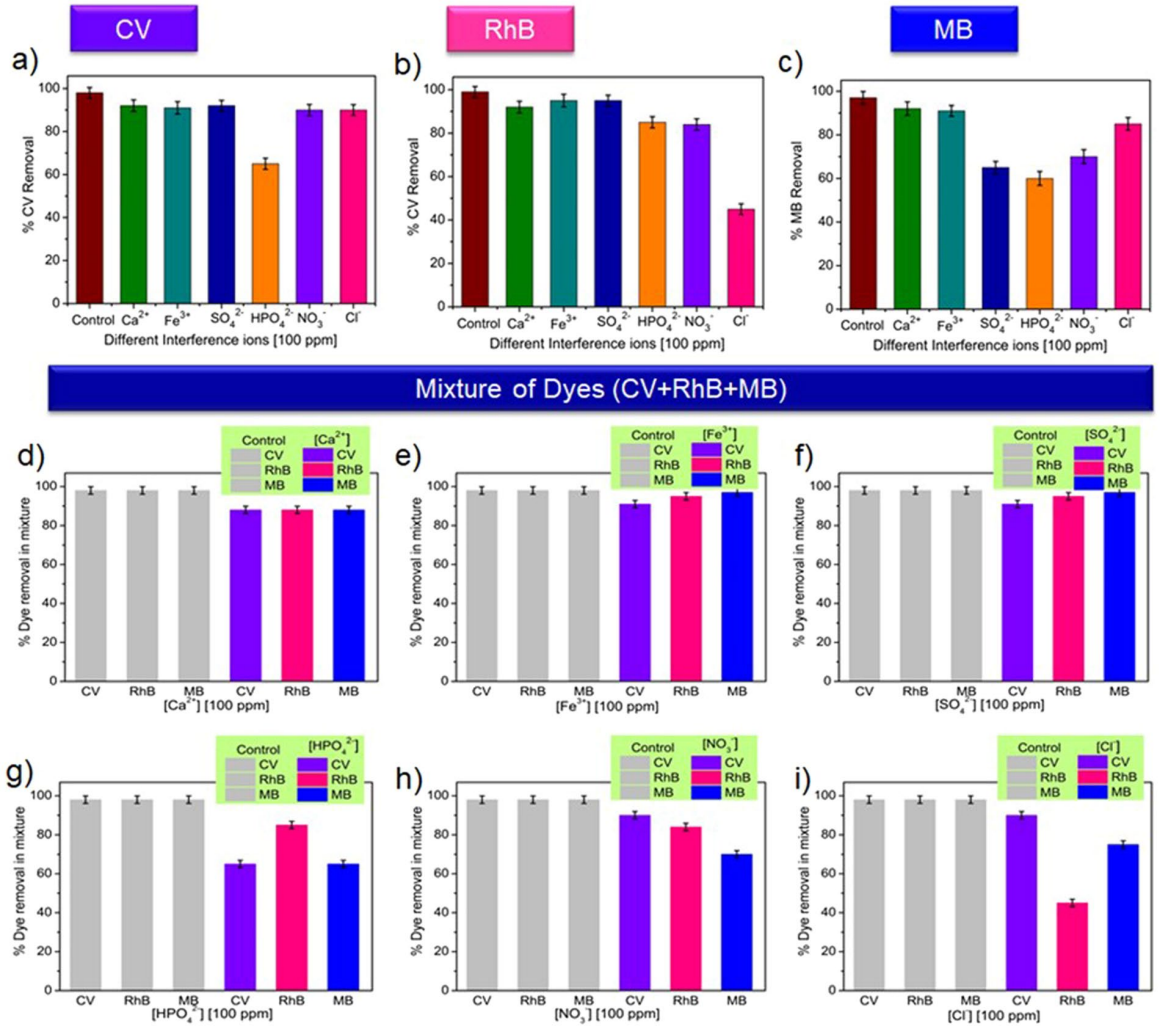
Effect of different interfering ions Ca^2+^, Fe^3+^, SO_4_^2−^, HPO_4_^2−^, NO_3_^−^, and Cl^−^) (100 ppm) on the decolorization efficiency of (**a**) CV (**b**) RhB, and (**c**) MB separately by wsGNS with respect to control; (**d**–**i**) Effect of different interfering ions Ca^2+^, Fe^3+^, SO_4_^2−^, HPO_4_^2−^, NO_3_^−^, and Cl^−^ (100 ppm) on the decolorization of mixture of dyes by wsGNS with respect to control.

To check the possible potentials for the practical-applications, the photodegradation efficiency of wsGNS was further been examined in the presence of various interfering ions^66^. Different interfering ions were mixed separately into separate dye solution and their photodegradation were carried out at same experimental conditions as discussed above. Figure 6(a–c) displayed the photodegradation efficiency of wsGNS towards CV, RhB, and MB in the presence of diverse interfering ions (100 ppm of Ca^2+^, Fe^3+^, SO_4_^2−^, HPO_4_^2−^, NO_3_^−^, and Cl^−^), suggesting that the photodegradation efficiency of wsGNS was not significantly affected even in the presence of many interfering ions. Only few ions were observed to affect the photocatalytic efficiency. Such as in the case of CV, only HPO_4_^2−^ intervened a bit, while in case of RhB it is only Cl^−^ ions and in the case of MB, the SO_4_^2−^, HPO_4_^2−^, and NO_3_^−^ ions interfered in its photodegradation. Similarly, for the mixture of the dyes (CV + RhB + MB) the effects of these interfering ions on the percentage degradation are shown separately in Fig. 6(d–i). The dyes degradations were monitored separately at their respective λ_max_ values of 589 nm, 554 nm, and 663 nm for CV, RhB, and MB respectively. In the described range the degradation of CV and MB in the mixture of dyes was not affected by most of the ions except by HPO_4_^2−^ while in case of RhB, Cl^−^ ions affected the degradation of RhB in the mixture of dyes. The delay in photodegradation of dyes in the presence of SO_4_^2−^, HPO_4_^2−^, NO_3_^−^, and Cl^−^ might be because of reaction of positive holes with these negative ions. Like there may be competition of negative ions with negative surface groups of wsGNS for the photodegradation of cationic dye molecules^66^.

### Treated Wastewater for the Growth of Wheat Plants

**Figure 7:**
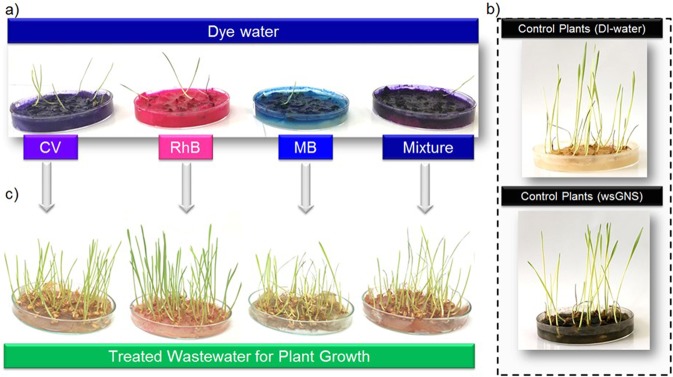
The effect of dyes, their mixture *verses* treated wastewater on the wheat plants was tested for the 15 days of germination; seeds treated with (**a**) dyes water and their mixture (CV + RhB + MB); (**b**) control (as in DI water and in wsGNS solution); *versus* the (**c**) treated wastewater of dyes and their mixture.

A simple eco-toxicological bioassay like the growth of plants from the germinated seeds was performed to check suitability and sustainability of overall photocatalytic process, related to the possible reuse of the treated waste water^88^. Influence of dyes and their mixtures before and after the photodegradation with wsGNS and control (only DI water and in wsGNS) were investigated on the growth of wheat plants as one of the most sensitive and fast growing plants. One day germinated wheat seeds were grown with pollutant water containing dyes, their mixtures and photodegraded treated wastewater and growth were observed after the 15 days of germination as shown in Fig. 7. The solutions of dyes and their mixtures before degradation showed a very-strong inhibition in the growth of wheat plants (Fig. 7(a)). However, the wheat plants were grown with photodegraded treated water showed the almost similar manner of growth compared to the control plants (treated with DI water and in wsGNS) (Fig. 7(b,c)). The growth observed in the case of wsGNS strongly advocated the non-toxic^24^ behavior towards the plant growth. The obtained results are in the favor concerning the safe uses of the treated wastewater. But for the edible plants, more precised and the thorough studies need to be taken care. Indeed, this practice can further lessen the overexploitation of natural water and could promote the reuse of treated wastewater to at least irrigate the playgrounds, parks, and gardens.

## Conclusions

In summary, environmentally benign isolation of wsGNS as an advanced photocatalytic material from the dirty-dangerous BC as a carbon source is being investigated here. The wsGNS manifested sunlight-driven highly effective photocatalytic activity for the almost complete photodegradation of the three chosen different organic dyes and their mixtures. In particular, the 99.9% photodegradation of the mixture of dyes was achieved within ~225 minutes. The mechanisms governing the prominent photocatalytic activity of wsGNS and reactive species responsible for dyes degradation were investigated by band gap measurements and active species scavenging experiments. The holes and hydroxide radicals were identified as active species responsible for the photodegradation. The breaking of the complex organic aromatic framework of the dyes into their smaller non-toxic versions were confirmed by the NMR analysis of the photodegraded products. Further, the sustainability of the overall process was to stand by the application of photodegraded wastewater from the pollutant dyes for the growth of the wheat plants, which show the remarkable results compared to the dye treated plants. As such, the use of wastewater for growing the wheat plants could relate to the practical sustainability of the treated water for its use in real-life applications and pragmatic solutions to environmental problems postured by dye-laden effluents.

## Materials and Methods

### Materials

Petrol engine soot was collected locally from the Jaipur city. MB was purchased from Sigma Aldrich, CV and RhB was purchased from LobaChem, Mumbai, India. All the experiments were performed using deionized water (DI water).

### Instrumentation

Structural characterization was performed through TEM and HR-TEM analysis with a Tecnai G^2^20 high-resolution TEM operating at a voltage of 200 kV. Samples for TEM/HR-TEM analysis were prepared by casting droplets of an aqueous solution of wsGNS onto a 400 mesh carbon-coated copper grid, followed by drying under 100 W table lamp for 12 h. The UV-Vis absorption analysis were done at room temperature with Perkin Elmer Lambda 35 spectrometer. XPS measurements was recorded in ESCA^+^ omicron nanotechnology oxford instrument. For FT-IR spectra measurements, BRUKER Vector22 IR spectrometer model with pressed KBr pellets was used. Raman spectra were done by WITEC model Raman spectrometer at wavelength 532 nm with an Ar^+^ laser. X-ray diffraction spectra were obtained at 25 °C (Cu Kα1, Kα2, Kβ radiation, with scan rate 2°/min) on a Pananalytical X Pert Pro Powder X-ray diffractometer model. PerkinElmer UV-Vis (NIR) spectrometer was used for carrying out UV-Visible DRS measurements. ^1^H NMR measurements were recorded on a JEOL ECS-400 (operating at 400 MHz, in D_2_O solvent).

### Synthesis of wsGNS

The isolations of the wsGNS is being the same as described earlier^25^.

### Photocatalytic experimental procedure

Three different types of dyes and their corresponding mixture were taken to examine the photocatalytic activity of wsGNS under direct sunlight illumination. All the photocatalytic experiments were carried out Jaipur, India in the month of May 2018. In a typical process stock solution of CV, RhB and MB of concentration, 20 ppm was prepared in DI water along with this the concentrations of all the three dyes in mixture maintained to 20 ppm in a conical flask for the photocatalytic degradation^65,67–69^. 0.3 mg mL^−1^ of wsGNS added separately to all the dyes solution (individual dyes as CV, RhB and MB and their corresponding mixture) and the solutions stirred for 30 min in the dark to reach the adsorption and desorption equilibration. During the photocatalytic experiments, fixed amount of photoreacted solutions were taken at regular time intervals. The collected solution was centrifuged and the supernatant was collected in a quartz cuvette for determining the dye concentration by using UV-Vis absorbance spectroscopy at wavelength 589 nm, 554 nm, and 663 nm for CV, RhB, and MB dyes respectively. For further characterization of the photodegraded products of different dyes by wsGNS, the complete photodegraded samples were collected and centrifuged, further the as collected supernatant was dried and dissolved in D_2_O to perform the NMR analysis.

### Germination

Wheat seeds (*Triticum aestivum*) were washed with distilled water and further soaked in tap water for the one day for germination.

### Seeds

Soaked moist seeds were placed in wet cotton cloth for one day. One-day-old sprouted wheat seeds were used for monitoring the growth under the controlled conditions (DI water and in wsGNS); in polluted dyes and their mixture versus the treated wastewater (water contained after the photocatalysis by wsGNS in the presence of sunlight). For the growth of the plants; one day germinated seeds were placed in petri dish containing the almost similar cotton sheets soaked with the equal amount (~10 ml for the first day) of (i) DI water; (ii) wsGNS; (iii) dyes and their mixture; and (iv) with their the respective treated wastewater. All the experiments were performed thrice for the 15 days, including the daily additions of ~3 mL of the each solution to check the growth of wheat plants.

### Regeneration analysis

The recovered wsGNS after photodegradation was regenerated with 0.01 M HCl solution repeatedly over ~10–12 times followed by washing with distilled water until the pH of the effluent become neutral. The regenerated sample after washing dried in the oven at 80 °C for the further use.

## Supplementary information

Soluble Graphene Nanosheets for the Sunlight-Induced Photodegradation of the Mixture of Dyes and its Environmental Assessment
