# Supplementary material for: Soluble Graphene Nanosheets for the Sunlight-Induced Photodegradation of the Mixture of Dyes and its Environmental Assessment
Source: Sci Rep. 2019 Feb 21;9:2522. doi: 10.1038/s41598-019-38717-1 (PMC6384933; doi:10.1038/s41598-019-38717-1)
Supplement: Supplementary file 1 — Soluble Graphene Nanosheets for the Sunlight-Induced Photodegradation of the Mixture of Dyes and its Environmental Assessment [file 41598_2019_38717_MOESM1_ESM.doc]

Electronic Supporting Information

**Soluble Graphene Nanosheets for the Sunlight-Induced Photodegradation of the Mixture of Dyes and its Environmental Assessment**

*Gunture,^†^ Anupriya Singh,^†^ Anshu Bhati,^†^ Prateek Khare,^†^ Kumud Malika Tripathi,*^‡^ and Sumit Kumar Sonkar*^†^*

^†^Department of Chemistry, Malaviya National Institute of Technology, Jaipur, Jaipur-302017, India

^‡^ Department of Bio-nanotechnology, Gachon University, Gyeonggi-do, South Korea

^*^Corresponding authors: E-mail: [sksonkar.chy@mnit.ac.in](mailto:sksonkar.chy@mnit.ac.in) [kumud20010@gmail.com](mailto:kumud20010@gmail.com)

Table of contents of supporting information

1. Figure S1:

2. Figure S2:

3. Figure S3:


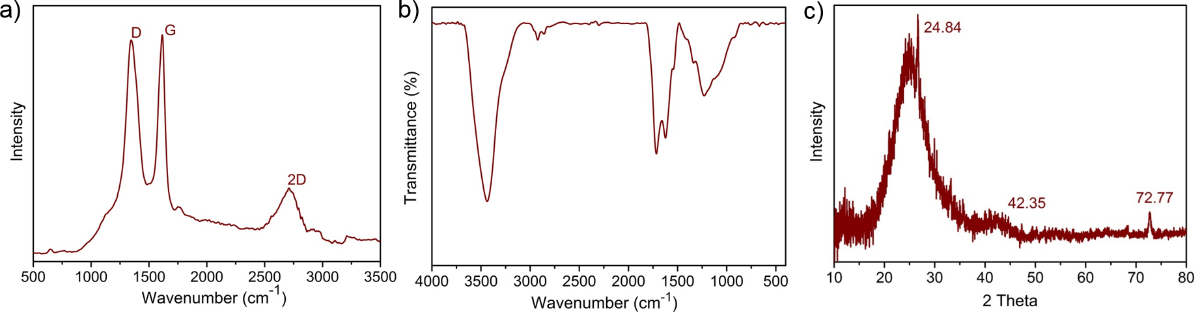


**Figure S1.** **(a)** Raman spectrum; **(b)** FT-IR spectrum; **(c)** XRD spectrum of wsGNS.

1. UV-Vis absorption study:

A detailed UV-Vis absorption study in Figure S2 (a-e) shows the relative changes in concentration of respective dyes and its mixture (decrease in the color intensity) as a function of time under the presence of sunlight. Figure S2 (a-c) show that there is a gradual decrement in absorption maxima with time at 589 nm for crystal violet (CV), 554 nm for rhodamine B (RhB) and at 663 nm for methylene blue (MB). Figure S2 (d) shows the absorption spectra of the mixture of dyes (CV+RhB+MB) which can be easily differentiated based on the three separate individual peaks in the mixture because of absorbance associated with CV, RhB, and MB with inset showing violet curve for CV, pink curve for RhB and blue curve for MB. Figure S2 (e) shows the gradual decrease in absorption maxima for different dyes in the mixture by wsGNS under sunlight irradiation and within 225 min mixture of has dyes has been decolorized as observed by UV-Vis absorbance study.


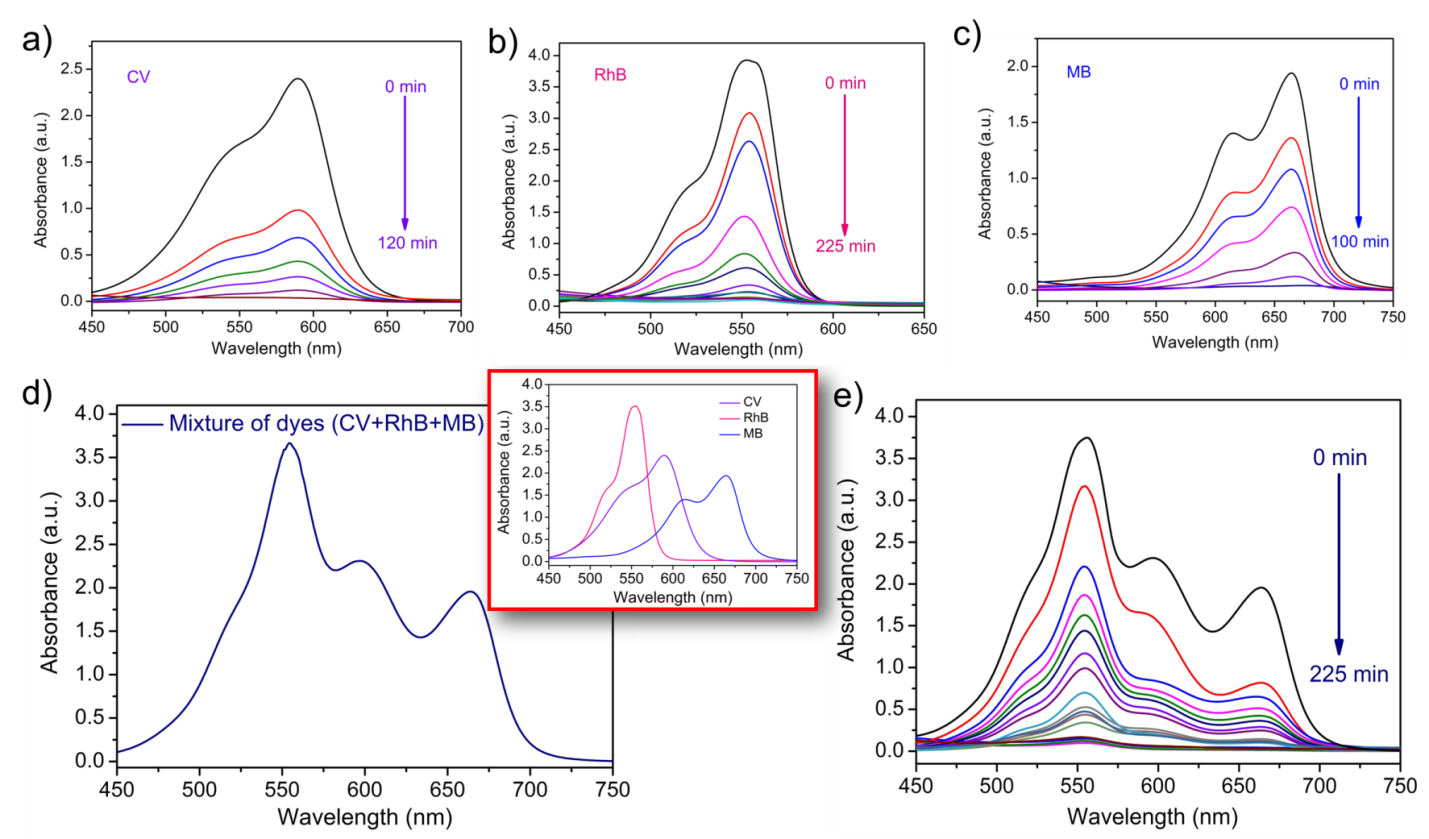


**Figure S2.** UV-Visible absorption spectra of **(a)** CV; **(b)** RhB; and **(c)** MB with time; **(d)** mixture of dyes (CV+RhB+MB) with inset showing violet curve for CV, pink curve for RhB and blue curve for MB; **(e)** UV-Visible absorption spectra of mixture of dyes with time by wsGNS under sunlight irradiation.

**
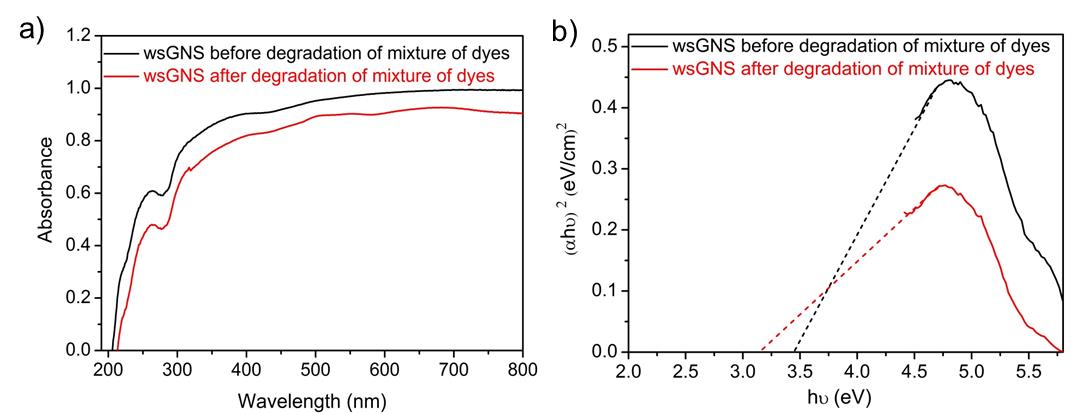
**

**Figure S3:** **(a)** UV−Vis DRS spectra of wsGNS before (black line) and after photodegradation (red line) and **(b)** Tauc plot of (αhυ)^2^ versus hυ of wsGNS before and after photodegradation of mixture of dyes.


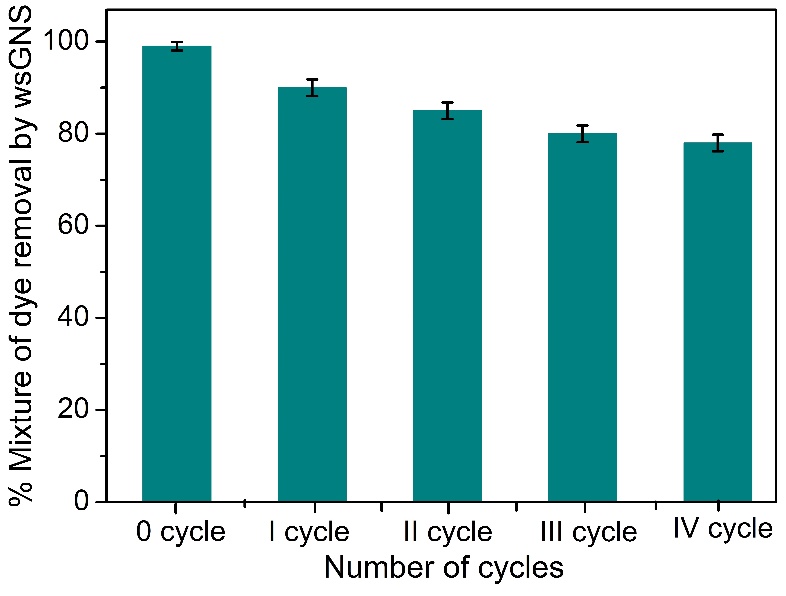


**Figure S4:** Photodegradation activity of wsGNS after the four cycles of recycling, testing in the case of the mixture of dyes.
